# Real-Time Water Quality Monitoring with Chemical Sensors

**DOI:** 10.3390/s20123432

**Published:** 2020-06-17

**Authors:** Irina Yaroshenko, Dmitry Kirsanov, Monika Marjanovic, Peter A. Lieberzeit, Olga Korostynska, Alex Mason, Ilaria Frau, Andrey Legin

**Affiliations:** 1 Institute of Chemistry, St. Petersburg State University, Mendeleev Center, Universitetskaya nab. 7/9, 199034 St. Petersburg, Russia; irina.s.yaroshenko@gmail.com (I.Y.); andrey.legin@gmail.com (A.L.); 2 Faculty for Chemistry, Department of Physical Chemistry, University of Vienna, Waehringer Strasse 42, 1090 Vienna, Austria; Monika.Marjanovic@univie.ac.at (M.M.); Peter.Lieberzeit@univie.ac.at (P.A.L.); 3 Faculty of Technology, Art and Design, Department of Mechanical, Electronic and Chemical Engineering, Oslo Metropolitan University, 0166 Oslo, Norway; olga.korostynska@oslomet.no; 4 Faculty of Science and Technology, Norwegian University of Life Sciences, 1432 Ås, Norway; alex.mason@nmbu.no; 5 Animalia AS, Norwegian Meat and Poultry Research Centre, P.O. Box 396, 0513 Økern, Oslo, Norway; 6 Faculty of Engineering and Technology, Liverpool John Moores University, Liverpool L3 3AF, UK; i.frau@2016.ljmu.ac.uk

**Keywords:** water quality, real-time monitoring, multisensor system, molecularly imprinted polymers, functionalised coating, microwave spectroscopy

## Abstract

Water quality is one of the most critical indicators of environmental pollution and it affects all of us. Water contamination can be accidental or intentional and the consequences are drastic unless the appropriate measures are adopted on the spot. This review provides a critical assessment of the applicability of various technologies for real-time water quality monitoring, focusing on those that have been reportedly tested in real-life scenarios. Specifically, the performance of sensors based on molecularly imprinted polymers is evaluated in detail, also giving insights into their principle of operation, stability in real on-site applications and mass production options. Such characteristics as sensing range and limit of detection are given for the most promising systems, that were verified outside of laboratory conditions. Then, novel trends of using microwave spectroscopy and chemical materials integration for achieving a higher sensitivity to and selectivity of pollutants in water are described.

## 1. Introduction

Water is one of the major natural resources for people. In 2012 it was declared that a safe water supply for every person is a crucially important task worldwide [1]. There are special water sustainability guides issued by the World Health Organization and regulated water quality standards [2]. The United Nations Sustainable Development Goals are the blueprint to achieving a better and more sustainable future for all-goal six specifically aims to ensure clean and accessible water. This, in turn, requires adequate water quality monitoring solutions specific to the situation. For example, summer 2019 was marked by catastrophic events in Norway, when more than 2000 people became sick, with more than 60 being hospitalized and 2 people dying as a result of an outbreak of *Campylobacter* and *Escherichia Coli* (*E. Coli*) that arose in the drinking water in Askøy, on the west coast of Norway. It is even more remarkable that this occurred in a country which has the status of being one of the countries with the highest quality of water in the world. The exact origin of the bacterial contamination that has caused this is still not confirmed, but the fact that there is a need for real-time monitoring of all drinking water reservoirs everywhere is undisputed. Therefore, this review examines various technologies that could meet these demands.

The conventional approach to qualitative water analysis assumes application of various chemical, physical and microbiological methods [3]. Most such methods demand specialized laboratories equipped with expensive and sophisticated scientific devices. Furthermore, highly qualified personnel are needed to operate such devices and special efforts and manpower must be spent for representative water sampling. More effective water quality control methods must be developed. Such methods should be fast, low-cost, with minimum automatic sampling and, ultimately, provide real-time results.

## 2. Current Situation with Online Water Analysis

**Figure 1:**
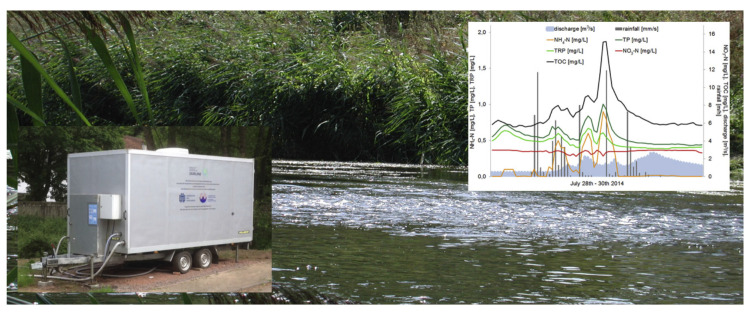
Mobile station for water quality monitoring and a sample of typical output of this station. Reprinted from Science of the Total Environment, Vol. 651, Angelika M. Meyer, Christina Klein, Elisabeth Fünfrocken, Ralf Kautenburger, Horst P. Beck, Real-time monitoring of water quality to identify pollution pathways in small and middle scale rivers, Pages 2323–2333, Copyright (2019), with permission from Elsevier.

A comprehensive description of the current situation with online water analysis can be found in [4]. Mobile chemical analysis stations were used in this work to monitor different water parameters. The systems were deployed in specially produced trailers (Figure 1) that were towed to the river banks.

The sensors, based on various principles, measured temperature, total phosphorus, pH and ammonium ions (by standard electrochemical sensors), dissolved oxygen, conductivity, nitrate ions and total organic carbon (by optical sensors). The measurements were performed by different stations in different locations. The measurement accuracy was within 10% for most measured parameters over 5 years of experiments in 35 locations along 25 small- and medium-size rivers. Such stations may likely improve our understanding of pollution types and pathways depending on water basins, seasonal factors and anthropogenic load. However, such stations cannot be considered as a practical instrument for wide-scale water quality monitoring due to their high cost, need for maintenance and significant power supply requirements.

Chemical sensors are attractive instruments for water quality analysis. The electrochemical or optical properties of such sensors may depend on the concentration of analytes in the water. Such sensors are already widely applied to the analysis of natural and potable water [5].

**Figure 2:**
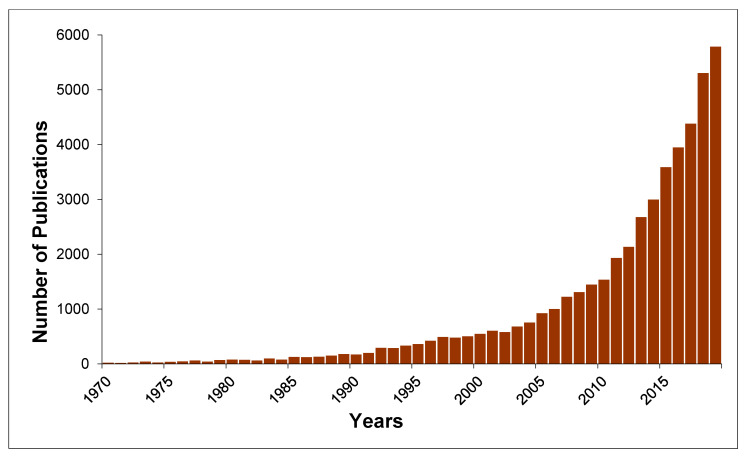
Number of publications published on the topic of the review in the last 50 years. Search keywords: “real-time water quality sensors”. Scopus (October 2019).

The growth of publication numbers in the field is shown in Figure 2.

## 3. Water Quality Monitoring Systems

The first sensor really suitable for water monitoring was the glass pH electrode, which appeared, in the present shape, along with a pH meter, around 1930. Since then, pH is a primary parameter of most water monitoring devices.

It is obvious, however, that multiple water parameters must be evaluated to responsibly judge its quality and multisensor systems should be applied for such purposes.

There have been multiple attempts to develop multisensor systems that could be applied for water quality control, e.g., [6,7]. However, the first efforts mostly dealt with laboratory water analysis rather than being applied in real-time, online mode.

For instance, a voltammetric sensor array with four electrodes (Au, Pt, Ir and Rh) served for multisite water quality monitoring at a water treatment plant [8]. The aqueous samples were taken at nine filtration steps, as well as before and after the complete procedure of water purification. The voltammetric data were processed by principal component analysis (PCA), revealing pronounced difference between raw, rapidly filtered and clean water. However, it was observed that the samples collected after treatment by several slow filters were close to the rapidly filtered water samples on the PCA scores graph. This can potentially be explained by the low efficiency of these filters. Therefore, it was concluded that a multisensor system approach is suitable for continuous control of water quality at treatment facilities, indication of the possible malfunctioning units and for checking the water status after maintenance. Significant influence of sensor drift and the necessity to compensate this drift was pointed out.

The system developed in [9] was designed to measure pH, temperature, dissolved oxygen, conductivity, redox potential and turbidity. This set of parameters is the most common one in water quality assessment since the sensors for these parameters can run in continuous mode. The whole set of sensors was mounted on aluminum oxide. All sensors were united into a single PVC body and their outputs were collected by the data acquisition system, which could also perform remote data transmission. The work suggests that the body might be dipped into water or even built into water flow. The device also included a set of electric valves and pumps for sampling, cleaning and calibration. The authors proposed that such a portable system can be suitable for water quality monitoring from different sources.

Chinese authors published a paper where a multisensor system was applied for the determination of several elements such as iron, chromium, manganese, arsenic, zinc, cadmium, lead and copper [10]. The device comprised three analytical detection systems: a multiple light-addressable potentiometric sensor (MLAPS) based on a thin chalcogenide film for simultaneous detection of Fe(III) and Cr(VI) and two groups of electrodes for detection of other elements using anodic and cathodic stripping voltammetry. The following detection limits were obtained: Zn—60 μg/L, Cd—1 μg/L, Pb—2 μg/L, Cu—8 μg/L, Mn—60 μg/L, As—30 μg/L, Fe—280 μg/L and Cr—26 μg/L. The authors recommended their method to determine metals simultaneously in seawater and wastewater; however, the possibility of application of this device for online analysis was unclear.

Potable water quality is of primary interest to people. Such type of water was studied in [11], using two sensor stations. The first one was used for detecting free chlorine with a precision of 0.5% and limit of detection (LoD) of 0.02 mg/L, as well as total chloride by colorimetric method with precision of 5% and LoD 0.035 mg/L. The second station used was a multisensor for detection of pH, redox potential, dissolved oxygen, turbidity and conductivity. Eleven different contaminants were injected into the flow of the studied liquid, namely pesticides, herbicides, alkaloids, E. coli, mercury chloride and potassium ferricyanide. It was demonstrated that the set of sensors produces a response for each type of contaminants. Unfortunately, the work does not report any data about the precision of such systems during long-term application.

**Figure 3:**
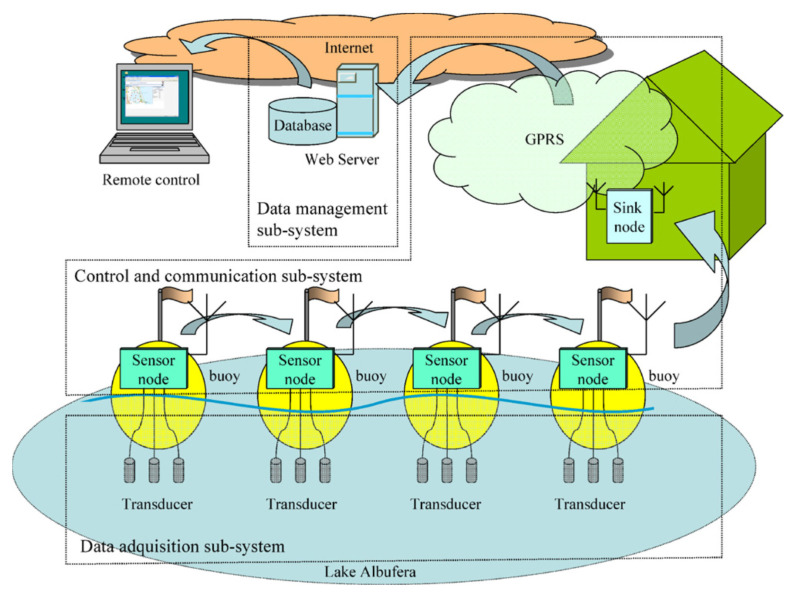
System for water quality monitoring. Reprinted from Talanta, Vol. 80, J.V. Capella, A. Bonastre, R. Ors, M. Perisa, A Wireless Sensor Network approach for distributed in-line chemical analysis of water, Pages 1789–1798, Copyright (2010), with permission from Elsevier.

Another device for online water analysis was suggested in [12]. Fourteen buoys were installed in a freshwater lake; each of them was equipped with three ion-selective electrodes detecting the concentration of ammonium ions along with nitrate and chloride. Wireless connection between buoys could be implemented using Global System for Mobile Communications (GSM) and General Packet Radio Services (GPRS) protocols. The data was accumulated in a single place. The data was accessible via the internet allowing real-time control of the system performance (Figure 3).

The authors reported daily drift of sensor readings of about 1.5 mV. Since the measurements were performed for only 7 days, the accumulated drift was not that significant. However, the influence of drift can be critical over longer periods of time.

Two multisensor systems were suggested in [13] for environmental monitoring of various contaminants. One was dealing with contents of ammonium, potassium, and sodium in a river with low anthropogenic load. The second system was installed in river water in a populated region and was designed for detection of heavy metals such as copper, lead, zinc and cadmium. The systems were also equipped with radio transmission devices. The system was tested just for 8 h, which is obviously too short a period for any serious conclusions about such a technology.

Wider research was performed in [14], which was conducted over a period of 12 months. A sensor array comprising eight conducting polymer sensors for gas phase analysis was used to detect abrupt changes in the wastewater quality. Free gas emanating from bubbled liquid in the flow cell with constant temperature was delivered to the sensor chamber for analysis. The results of field tests at the water treatment plants using automatic systems produced water quality profiles and displayed the possibility of determining both random and model contaminants. This approach showed high sensitivity and flexibility and low dependence on long-term drift, daily oscillations, temperature and humidity. It must be noted, however, that the described experiments were carried out not at a real water treatment plant, but in a pilot system. Thus, the diversity of its performance may not be representative for real-world conditions. Besides, the idea to follow water quality via headspace analysis is obviously limited: it is impossible to follow contaminants which are not volatile enough.

## 4. Application of Biosensors and Optical Sensors for Water Quality Assessment

Biosensors were also used for water quality control, though quite a few of them were applied for online flow analysis. Pesticides were the main target of biosensors.

A system of biosensors capable of determining dichlorvos and methylparaoxon in the water was suggested in [15]. The systems consisted of three amperometric biosensors based on various AChE (acetylcholinesterase) enzymes. These enzymes were immobilised in a polymeric matrix onto the surface of screen-printing electrodes. The enzymes solutions were deposited over the electrode surface and irradiated by light, inducing photo polymerisation of the azide groups in the molecules. Such a sensor array was built into a flow system permitting automatic analysis. Bottled and river water was studied. The concentration of pesticides was detected in the ranges 10^−4^–0.1 μM for dichlorvos and 0.001–2.5 μM for methylparaoxon. Solely spiked samples were considered; therefore it is necessary to further verify performance of such a system in online mode.

Another work [16], reports on using Pt electrodes instead of screen-printed ones and self-made carbon paste was applied as a sensing layer. The paper implies that such a procedure may improve sensitivity of the substrate for some of the immobilized enzymes. The total number of biosensors in the array was eight. The ultimate aim of this research was not a quantification of pollutants but a global evaluation of water quality. It is doubtful though, if such a quality can be precisely determined by biosensors, which are highly selective to the main substance and would exhibit low cross-sensitivity to many other analytes present in the natural water.

One more attempt to evaluate global water toxicity by biosensors is described in [17]. The online toxicity monitoring system employed sulfur oxidizing bacteria (SOB) and consisted of three reactors. No toxicity changes in the natural flow water were observed over a period of six months. When the flow was spiked with diluted pig farm waste, the activity of sulfur oxidizing bacteria decreased by 90% in 1 h. The addition of 30 μg/L of nitrite ions or 2 μg/L of dichromate ions resulted in full degradation of sulfur oxidizing bacteria activity in 2 h. Thus, the sensitivity of the system to both inorganic and organic pollutants was demonstrated. It must be noted that one or two hours is a rather long period of time for detecting acute contaminations; functionality of the system could be regained only by introducing a new portion of bacteria, which significantly impairs real-time, online application of such device.

Optical sensors were also recently applied for water quality analysis, however, these are mostly discrete sensors, though tuned sometimes for integral parameters such as water color, turbidity or even COD and BOD. Discrete sensors were used to determine chlorophyll in the seawater on the basis of its fluorescence [18], for evaluation of water opacity and color evolution by LED [19], for analysis of water turbidity and color in online mode [20] as well as for determination of heavy metal ions [21].

## 5. Biomimetic Approaches for Sensing Water Quality

### 5.1. Chemical Sensors for Sensing in “Real-Life Environments”

Biosensors reveal exceptional selectivity and often sensitivity, but usually are limited in terms of ruggedness and technical applicability in non-physiological conditions. One way to overcome this is to implement bioanalogous selectivity into systems that are able to withstand harsh and non-physiological conditions, so-called biomimetic systems [22]. Molecularly imprinted polymers (MIPs) are a promising example of such synthetic materials [23], since they are robust due to their highly cross-linked nature. Furthermore, they come at much lower costs than natural materials and provide longer storage and use periods. MIPs can also be produced for molecules that cannot be detected by natural receptors [24].

**Figure 4:**
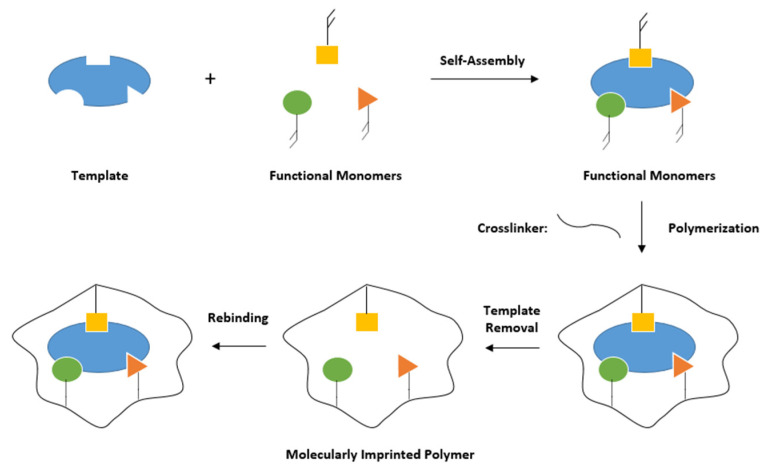
Schematic overview of molecular imprinting.

MIPs are generally synthesized by co-polymerization of functional and cross-linking monomers in the presence of a template (see Figure 4). Initially, a complex forms between functional monomers and the template through weak, noncovalent interactions (mainly hydrogen bonds, Van-der-Waals or π-π interactions), followed by polymerisation with cross-linking monomers to form a rigid, three-dimensional polymeric network. Removal of the template leads to recognition sites (cavities) within the polymer that are complementary to the target molecule in size, shape, and chemical functionality and are suitable to selectively rebind the analyte [25].

Except for MIPs, target recognition can also be obtained using other strategies. Aptamers, for example, are single-stranded RNA or DNA oligonucleotides, whose tertiary structure selectively binds their target molecules [26]. Another option is whole-cell-based sensors, which were also already applied to real wastewater samples [27,28]. In this case, mammalian cells were used for detecting harmful and toxic compounds, because their closeness physiology is close to that of humans. Although this strategy may not be regarded biomimetic in the strict sense of the word, it provides direct information about the overall toxicity of samples rather than detecting or quantifying one specific substance. When applying this method, unknown or new chemicals and pollutants may be detected. Kubisch et al. used rat myoblast cells in combination with a commercially available multiparametric readout system to measure impedance (morphological integrity) and two metabolic parameters—acidification and respiration—to investigate the overall toxicity and bioavailability of substances in water samples [27]. This was achieved by using three different types of electrodes on a single chip surface: impedance, pH and oxygen (CLARK) electrodes. After testing different test compounds, including metal ions and neurotoxins, the system was exposed to real wastewater samples. It responded to different contaminants and was indeed suitable for monitoring unknown, harmful compounds in water. Similarly, the group of C. Guijarro applied rat liver cells in a whole-cell-based sensor system to monitor environmental contaminants, including an insecticide and a flame retardant, in water samples using the same analyzing system [28].

The aforementioned advantages of MIPs make them an attractive tool for different applications, such as solid phase extraction, drug targeting, development of sensors for various types of analytes, and environmental monitoring. Although the number of publications concerning MIP-based sensors is rising, only a small amount is actually applied to real-life environments or complex matrices. This part of the review will provide an overview of chemical sensors that were tested in (real-life) water samples with a special focus on receptor layers based on MIPs.

### 5.2. MIP-Based Sensors for Water Analyses

**Figure 5:**
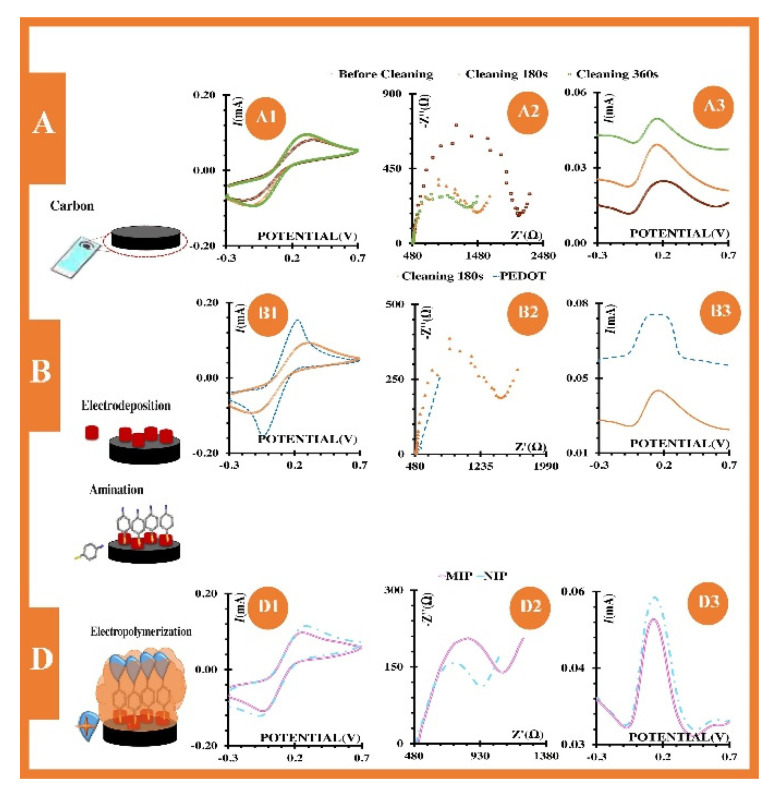
Construction principle and setup of chloramphenicol sensor. Reproduced from [30] with permission © Elsevier B.V. 2018.

In 2018, Ayankojo et al. introduced a sensor system capable of detecting pharmaceutical pollution in aqueous solutions [29]. They chose amoxicillin as the model analyte and implemented a hybrid MIP, consisting of organic and inorganic components, on the gold surface of a surface plasmon resonance (SPR) transducer. The hybrid MIP film was synthesized by applying the sol-gel technique and using methacrylamide as organic monomer and vinyltrimethoxysilane as inorganic coupling agent to form a stable and rigid polymeric network. Sol-gels have a highly porous structure and recognition sites are usually formed in a more ordered way. This results in enhanced sensitivity and faster sensor response times. Rebinding experiments of the amoxicillin MIP in phosphate-buffered saline (PBS) and tap water revealed an imprinting factor of 16 compared to the nonimprinted polymer (NIP) and a limit of detection LoD = 73 pM. Furthermore, the MIP responded almost exclusively to its target analyte thus exhibiting utmost specificity. In the same year, the group of Cardoso also developed a sensor for detecting chloramphenicol, an antibiotic used in fish farms [30] (see Figure 5). The corresponding MIPs were electro-polymerized on screen-printed carbon electrodes.

Impedance and square wave voltammetry (SVW) in both electrolyte solution and water from a fish tank served to investigate the performance of the recognition element. In case of impedance measurements in electrolyte solution, sensor characteristics were linear in a concentration range from 1 nM to 100 µM, achieving an LoD = 0.260 nM; SVW yielded similar characteristics and an LoD = 0.653 nM. In real-life samples—water from a fish tank—sensors responded linearly down to 1 nM and achieved an LoD of 0.54 nM and 0.029 nM for impedance and SVW measurements, respectively. These results suggest that there is no significant impact on sensor behavior when switching from standard solutions to real water samples leading to reproducible and sensitive sensor characteristics over five orders of magnitude down to 1 nM.

**Figure 6:**
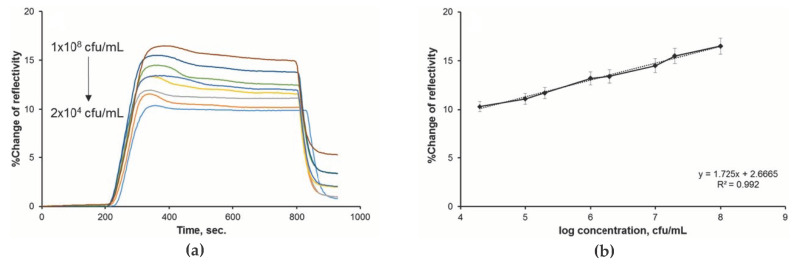
Sensor responses for faecal indicators, showing the (**a**) % change of reflectivity by time and (**b**) its linear correlation with the concentration. Reproduced from [31] with permission © Elsevier B.V. 2019.

The real-life feasibility of MIP-based sensor systems were also demonstrated in case of detection of faecal contamination of seawater samples [31]. MIP nanoparticles were fabricated for sensing *Enterococcus faecalis* (*E. faecalis*) serving as faecal indicator to assess the water quality. Such MIP nanoparticles have the advantage of a higher surface-to-volume ratio, which means that the resulting cavities or binding sites are easier to access by target analytes [5]. *E. faecalis*-imprinted nanoparticles demonstrated good SPR sensor performance in aqueous and real seawater samples:

As can be seen from Figure 6, changes in refractive index were linear in a concentration range from 2 × 10^4^–1 × 10^8^ CFU/mL covering four orders of magnitude with a limit of detection of 1.05 × 10^2^ CFU/mL. Selectivity studies with structurally similar bacteria revealed higher affinity of MIP nanoparticles towards the imprinted analyte compared to the other competitors. Selectivity coefficients for *E. coli, Staphylococcus aureus* and *Bacillus subtilis* were as follows: 1.38, 1.25 and 1.37.

Khadem et al. fabricated an electrochemical sensor for detecting diazinon, an insecticide, based on a modified carbon paste electrode combined with MIPs and multi-wall carbon nanotubes (MWCNTs) [32]. Using the latter modifier improves conductivity, whereas MIPs offer the necessary sensitivity towards the template molecule. After optimizing electrode composition, the method was first validated in aqueous standard solutions. SVW measurements revealed that the MIP showed much higher affinity to the analyte than the reference, the nonimprinted polymer; the system achieved linear performance in the concentration range from 5 × 10^−10^ to 1 × 10^−6^ mol/L with a calculated LoD = 1.3 × 10^−10^ mol/L. Furthermore, it was considerably more selective to the analyte than to other tested substances (ions and other pesticides). To investigate the applicability of the system to real biological and water samples, different amounts of diazinon were spiked to urine, tap and river water. In all these cases the sensors detected the target analyte with high recovery rates (>92%). This work demonstrates the use of MIP-based sensors in real-life samples and environments without the need of special sample pretreatment or preconcentration steps.

**Figure 7:**
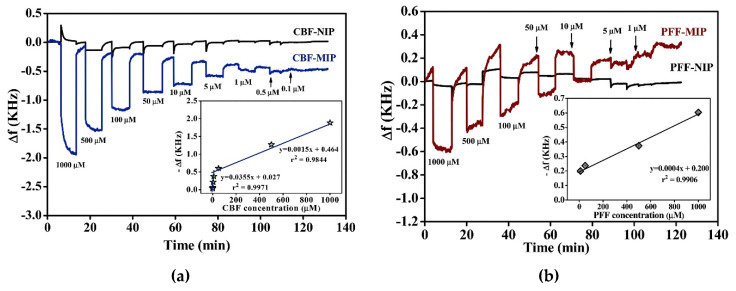
Frequency measurements of MIP- and NIP-coated QCMs for detection of (**a**) CBF and (**b**) PFF at different analyte concentrations. Reproduced from [33] Creative Commons License CC BY-NC-ND 4.0.

Another example for pesticide detection is presented in the work of Sroysee et al. [33]. They developed an MIP-based quartz crystal microbalance (QCM) sensor for quantification of carbofuran (CBF) and profenofos (PFF). For that purpose, an in-house-developed dual-electrode system was used, where one electrode pair served as reference with the upper electrode being coated with the NIP. Doing so offers the advantage of measuring MIP and NIP simultaneously under the same conditions. Applying the bulk imprinting method, MIPs for PFF were based on polyurethanes whereas CBF MIPs were synthesized using acrylic monomers. Frequency measurements of MIP- and NIP-coated QCMs are shown in Figure 7.

One can clearly see that both CBF and PFF MIPs led to linear sensor responses between 0.5–1000 µM and 5–1000 µM for CBF and PFF, respectively, whereas the frequency signal of the NIP stayed more or less constant.

Polycyclic aromatic hydrocarbons (PAH) are organic compounds which consist of at least two condensed aromatic rings. They are released into the environment through incomplete combustion of organic materials and considered to be mutagenic and carcinogenic. They usually occur in mixtures and their concentrations in air, water and sediments can be very low. Therefore, detection systems for PAH analysis need to be sensitive and selective. In particular, fluorescent sensors based on MIPs have gained in popularity due to their advantageous properties, such as high specificity, sensitivity and reversibility. Having a linear concentration dependency and low LoDs, those sensors seem to be quite promising for rapid detection of PAHs in aqueous solutions [34].

Sensors for the detection of nutrient components have been developed as well. For example, Warwick et al. reported a detection system based on MIPs combined with conductometric transducer for monitoring phosphates in environmental water samples [35]. Previous studies demonstrated that N-allylthiourea was the appropriate monomer for phosphate recognition [36]. The thiourea-based MIP was first optimized in terms of the optimal cross-linking monomer and ideal ratio of functional monomer to template (phenylphosphonic acid). Of all cross-linking monomers that were tested, ethylene glycol dimethacrylate (EGDMA) had the highest capacity of retaining phosphate as well as a monomer to template ratio of 2:1. After optimization, MIP membranes were integrated into the conductometric measuring cell. Selectivity tests in laboratory samples revealed no cross-talk to other ions, nitrate and sulfate. Both types of samples—standard and real-life ones—led to a linear increase in conductance with increased phosphate concentrations. In wastewater samples spiked with different amounts of potassium phosphate, the system allowed for LoD and LoQ values of 0.16 mg/L and 0.66 mg/L, respectively. The maximum acceptable amount of phosphate in wastewater is 1–2 mg/mL. The implemented sensor system with a linear range from 0.66 to 8 mg/mL therefore seems very promising for detecting small amounts of phosphate in environmental samples.

### 5.3. MIP-Based Sensors for On-Site Applications

**Figure 8:**
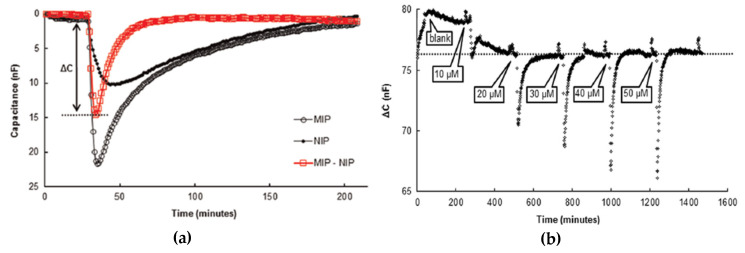
(**a**) Capacitance (nF) vs. time (min) of MIP, NIP and the differential signal ΔC (MIP-NIP). (**b**) Differential signal (ΔC in nF)) for different concentrations of metergoline vs. time (min). Reproduced with permission from [37] © Elsevier. MIP: molecularly imprinted polymers, NIP: nonimprinted polymers.

For a pollutant sensor to be applicable on-site in real-life environments, it needs to be selective, reusable, robust and able to withstand harsh conditions. In 2015, Lenain et al. [37] reported a sensor that met all those criteria: it consists of spherical MIP beads deposited onto the electrodes of a capacitive transducer via electro-polymerization. Corresponding MIP beads for detecting metergoline—a model analyte for small molecules such as insecticides and pharmaceuticals—were synthesized through emulsion polymerization. Different concentrations of metergoline in PBS buffer were measured with the difference in capacitance between MIP and NIP representing the specific binding of the analyte as depicted in Figure 8. As shown in the figure, capacitance decreased with increased concentrations (10–50 µM). Furthermore, the system was able to regenerate itself without adding regeneration buffer, demonstrating reusability of the sensor.

The sensor was also able to withstand harsh environments and achieved both a low LoD (1 µM) and low cross-selectivity. All these results suggest its suitability for monitoring pollutions originating from substances like pesticides or antibiotics in water samples (rivers, seawater) on-site.

Another example of a method suitable to the monitoring of contaminants in water *in situ* was introduced by Cennamo et al. [38]. It consists of an SPR sensor with an integrated plastic optical fiber (POF) combined with MIPs for detecting the model analyte perfluorobutanesulfonic acid (PFBS). With an LoD of 1 ppb, an interface software and the ability to connect to the internet directly, the SPR-POF-MIP technique is inherently suitable to detect small concentrations of different toxic or harmful compounds in real water samples *in situ*. Other advantageous features such as its reduced size, robustness and remote sensing abilities are further key factors for industrial applications of MIP-based sensor systems.

### 5.4. Mass Production of MIP-Based Sensor Systems

To prove that MIP-based sensors are inherently suitable for mass manufacturing, Aikio et al. developed a low-cost and robust optical sensor platform based on integrated Young interferometer sensor chips, where waveguides were fabricated on top of a carrier foil via roll-to-roll manufacturing techniques [39]. For chemical sensing of melamine, MIPs were used as recognition materials, whereas for biosensing of multiple biomolecules, sensor chips were functionalized with antibodies. In case of melamine sensing, the change in phase depended on the analyte concentration: Sensor responses increased with higher concentrations. Furthermore, the reference (NIP) led to much lower phase changes compared to sensor responses of the MIP. However, injection of higher concentrations (>0.5 g/L) led to saturation effects. For multianalyte biosensing, the sensor chip was functionalised with antibodies for C-reactive protein (CRP) and human chorionic gonadotropin (hCG) via inkjet printing. The results indicated that the Young interferometer bearing a specific antibody indeed selectively detected its corresponding protein. This work demonstrated the use of large-scale production techniques to develop a cost-efficient and rugged sensor system.

## 6. Functionalised Electromagnetic Wave Sensors

### 6.1. Microwave Spectroscopy and Water Analysis

Using electromagnetic (EM) waves at microwave frequencies for sensing purposes is an active research approach with potential for commercialization. This novel sensing approach has several advantages, including noninvasiveness, nondestructiveness, immediate response when the EM waves are in contact with a material under test, low-cost and power. Microwave spectroscopy provides the opportunity to guarantee continuous monitoring of water resources and intercept unexpected changes in water quality [40].

During the last 3 decades, microwave spectroscopy for liquid sensing has been investigated [41]. A water sample is placed in direct contact through a sensing structure and measured in real-time using an EM source (such as a vector network analyzer). The EM field interacts with the sample under test in a unique manner, depending on the polarization of water molecules and other compounds in the water samples, which produces a specific reflected or transmitted signal. The spectral response at specific frequencies depends on the conductivity and permittivity of the material under test [42]. Considering the variability of the sensing structures, the most successful experiments for detecting water quality were obtained using resonant cavities and planar sensors, due to the practicability of measuring a liquid sample.

Cavities resonate when the wavelength of the excitation within the cavity coincides with the cavity’s dimension [43]. They enable noncontact, real-time measurements, as liquid samples in plastic or glass containers with known dimensions and properties can be inserted into the cavity. Several experiments have shown the resonant cavity ability to detect the presence and concentration of various materials. Specifically, cylindrical cavities were used to determine water hardness [43], nitrates [44], silver materials [45] and mixtures such as NaCl and KMnO_4_ [46]. A rectangular resonant cavity was developed and tested for measuring pork-loin drip loss for meat production industry applications [47]. Another rectangular cavity was designed and tested for monitoring water quality, specifically the presence and concentration (>10 mg/L) of sulphides and nitrates [48].

**Figure 9:**
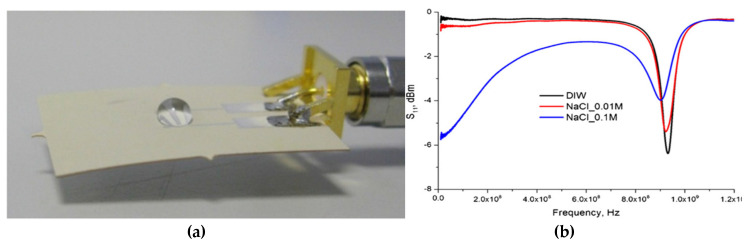
(**a**) Microwave flexible sensor with 20 µL of water sample placed the silver IDEs and (**b**) its output sensing response comparing deionized water (DIW), and 0.01 and 0.1 M of NaCl. Reproduced with permission from [49] © 2020 IOP Publishing Ltd.

During the last few years, several planar microwave sensors with different conformations have been developed and tested for differentiate compositions of water for both qualitative and quantitative concentration measurements [49,50,51,52,53]. Between planar sensing structures, Korostynska et al. [49] confirmed the action of a novel planar sensor with a sensing element consisted of interdigitated electrodes (IDE, also defined as interdigital by other researchers) metal patterns (silver, gold and/or copper) for water analysis observing changes in the microwave part of the EM spectrum analysing 20 µL of deionized water (DIW), KCl, NaCl and MnCl at various concentrations (Figure 9a,b). Then, Mason et al. [50], Moejes et al. [51] and Frau et al. [52] demonstrated the ability to detect respectively Lincomycin and Tylosin antibiotics, *Tetraselmis suecica* and lead ions (Pb^2+^) using gold (Au) eight-pair IDE sensors.

### 6.2. Progresses and Challenges in Microwave Spectroscopy

Microwave spectroscopy is an attractive option for detecting changes in materials in a noninvasive manner, at low cost with the option of portability and rapid measurements. This strategy, however, suffers from a deficiency of specificity, related to low sensitivity (ΔdB related with small changes in material changes) and selectivity (diverse spectral response for similar pollutants) [48,53]. Some of the disadvantages are also related to the capability to detect minor changes in the water sample which are not related to the changes in the target analyte, such as temperature and density [54].

There has been increasing research and development on understanding and improving the sensing performance of microwave spectroscopy for a deeper analysis of specific pollutants and small concentration changes related to them. Also, changes in the shape pattern of the sensing structure are not able to improve the performance of required sensitivity and selectivity of pollutants [55]. The bigger problem remains the detection of more than two pollutants at low concentrations.

**Figure 10:**
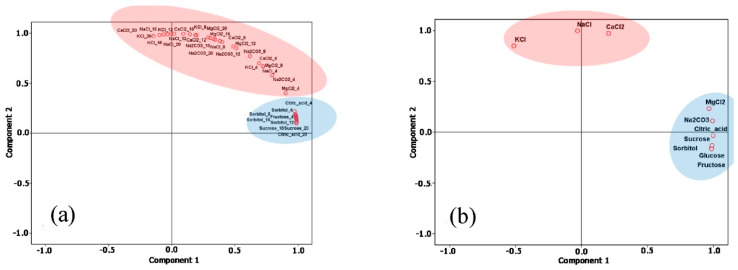
Discrimination between sugars and organic acid (blue oval) and salts (red ovals) using principal component analysis (PCA). (**a**): magnitudes of the transmission coefficient (S21) between 2.3 and 2.6 GHz; (**b**): amplitude of S21 at the resonance frequency. Reproduced with permission from [57] © Elsevier.

Novel strategies are being adopted to improve sensitivity and selectivity using microwave spectroscopy, but no one has yet demonstrated the feasibility of distinguishing low concentrations of similar substances in water. Amirian et al. [56] simulated the feasibility of distinguishing between pure liquid materials, such as ethanol, ammonia, benzene and pentene using a novel sensor design and mathematical approach, reaching higher sensitivity and noise reduction. Harnsoongnoen et al. [57] demonstrated the discrimination of organic and inorganic materials using planar sensors and principal component analysis (PCA). Magnitude spectra at 2.3–2.6 GHz were able to measure specific concentrations of sucrose, glucose, NaCl and CaCl_2_ citric acid between others, generating linear and nonlinear prediction models, correlating the transmission coefficient (S_21_) and R^2^. PCR method was used to divide samples into two groups using the S_21_ magnitude: sugars and organic acids (blue oval) and salts (red oval) in Figure 10.

The following year Harnsoongnoen et al. [58] proposed a novel approach to discriminate between phosphorus and nitrate using the transmission coefficient and the ratio between the resonance frequency and the frequency bandwidth at the magnitude of 10 dB. This proposed method offers high sensitivity for both nitrate and phosphates, but not yet the required specificity.

**Figure 11:**
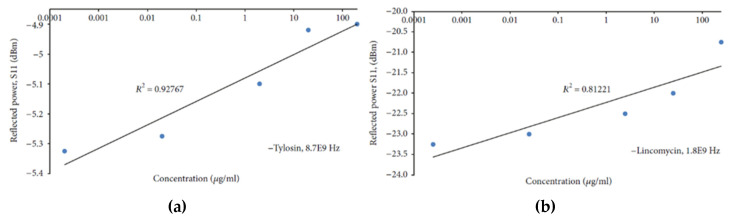
(**a**) Dependence of the S_11_-transmitted signal on tylosin and lincomycin at respectively, 8.7 GHz and (**b**) 1.8 GHz. Copyright © [50] under the Creative Commons Attribution License.

Other researchers are using machine learning features for selecting and distinguishing a target material [44] The developed model has been able to estimate the presence of nitrate in deionized water above the threshold, but it has not been able to quantify the precise concentration. Mason et al. [49] adopted a combined sensor approach using microwave analysis, combined with optical and impedance measurements for a more selective and sensitive determination of antibiotics, tylosin and lincomycin at different frequencies (at 8.7 and 1.8 GHz respectively) reaching high sensitivity (Figure 11a,b). Specifically, the selected planar microwave sensors were able to detect 0.20 µg/L of lincomycin and 0.25 µg/L of tylosin, a common concentration found in both surface and groundwater.

### 6.3. Microwave and Materials Integration

Further improvement in sensitivity and selectivity are essential steps for water quality sensing, especially in complex mixtures [59]. A recent attractive approach is the integration of sensing materials onto the sensing structure, which has been experimented with the use of electrochemical impedance spectroscopy (EIS) and IDE sensors [60] using diverse coating thicknesses and for microwave gas sensing [61]. Planar sensors are an attractive option for the implementation of materials such as thin and thick films or microfluidic structures [53].

**Figure 12:**
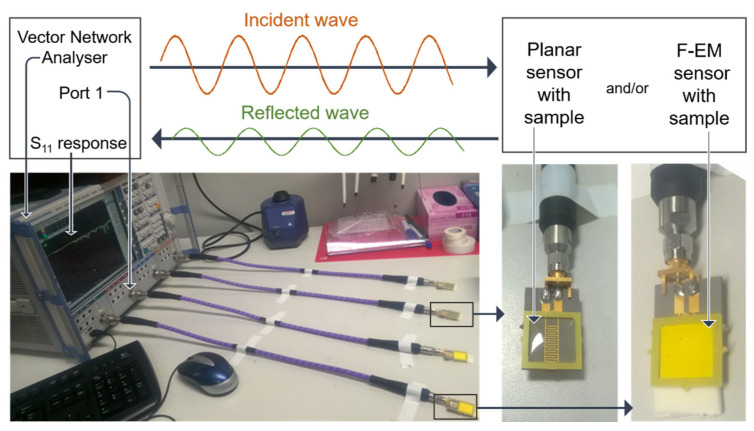
Scheme of the microwave interaction generated from a vector network analyser (VNA) which interact with a planar sensor (uncoated or functionalized with a coating, defined f-EM sensor) and a water sample placed onto it which generate a specific reflected signal (S_11_) at selected frequencies. Reproduced from [65], Creative Commons Attribution 4.0 International License.

The synergy between microwave sensing technology and chemical materials provides interesting advantages in the field of quality monitoring for adapting this method to a specific purpose and it is consequently a promising area of research and development. The integration of specific materials in the form of thin and thick films onto planar sensors has been recently recognized as a novel and attractive approach for reaching higher selectivity, sensitivity and specificity for a selected material under tests using microwave and impedance spectroscopy [59,62,63,64]. The principle is based on two processes: the sensing process, where the target analyte interacts, via physical or chemical interaction, with the material on the sensing structure, and the transduction process, where the interaction between EM waves, material and sample generate a particular signal [61]. A vector network analyzer is used as a microwave source and can be configured with one or two ports. One-port configuration (S_11_ measurement) measures the reflection coefficient of a material under test, which depends on how much the incident wave propagates through, or is reflected by the sample. Two-port configuration (S_21_ measurement) allows for measuring the transmission coefficient, which depends on how much EM power propagates from one port (port 1) through the sample and is received at the second port (port 2). This configuration allows the determination of both transmitted and reflected signals. S-parameters vary with frequency. By functionalizing planar sensors with certain sensitive materials using screen-printing technology, which are defined functionalized electromagnetic sensors (f-EM) sensors, it is possible to obtain the desired sensitivity and/or selectivity to one or more specific analyte in water obtaining an immediate specific response as the sample is placed onto the microwave sensor (uncoated or f-EM sensor) [65] (Figure 12). Accordingly, such work has the foundations for developing new methods, based on EM sensors and functional chemical materials, capable of determining specific chemicals in water, both qualitatively and quantitatively [65]. The sensing response as S_11_ can be determined by direct contact between the material and the analyte under test, which changes the permittivity of each component, and the consequent overall complex permittivity changes. The improvement can be associated with the increase of material thickness as well as the composition itself [66].

### 6.4. F-EM Sensors for Toxic Metals Analysis

Among the toxic elements, copper (Cu) and zinc (Zn) belong to the most common contaminants associated with mine wastes, which pollute water resources. Progress has been made in the last decade in developing chemo-sensors using mostly optical and electrochemical techniques. These are able to recognize specific metal ions using synthetic, natural and biological receptors [67], zeolites, inorganic oxides [68], organic polymers, biological materials [69], carbon-based materials [70] and hybrid ion-exchangers [71]. The interaction between the material and metal ions is the base for accredited optical and electrochemical sensing systems for detecting small concentrations in water

Currently, no certified method can guarantee real-time monitoring of toxic metals in water. Microwave sensing technology is promising for facing this challenge, although new strategies must be developed for obtaining more specific response. The integration of certain sensitive materials onto the planar sensing structure can be used to obtain the desired sensitivity and/or selectivity to specific analytes in water. Among these functional chemical compounds, inorganic oxide compositions are considered advantageous owing to their strong adsorption and rapid electron transfer kinetics [68,72]. Inorganic materials have attracted considerable attention owing to their low cost, compatibility and strong adsorption of toxic metal ions [69]. For instance, zinc oxide (ZnO) nanoparticles are well-known for strongly adsorbing Cu and Pb ions [73].

Frau et al. [74] laid the foundations for developing new methods, based on EM sensors and functional chemical materials, capable of determining in real-time metal content in mining-impacted waters, both qualitatively and quantitatively. Specifically, integrating planar electromagnetic wave sensors operating at microwave frequencies with bespoke thick film coatings was proven feasible for monitoring of environmental pollution in water with metals caused by mining [74]. A recently developed f-EM sensor based on L-CyChBCZ (acronym for a mixture based on l-cysteine, chitosan and bismuth zinc cobalt oxide) was tested by probing a polluted water sample spiked with Cu and Zn solutions using the standard addition method. The S_11_ response has shown linear correlation with Cu and Zn concentration at three resonant frequencies. Especially at 0.91–1.00 GHz (peak 2) the f-EM sensor shows an improvement for Cu detection with an improvement in sensitivity, higher Q-factor and low LoD compared with an uncoated (UNC) sensor, shown in bold in Table 1 [74].The sensor was able to detect Cu concentration with a limit of detection (LoD) of 0.036, just above the environmental quality standards for freshwater (28–34 µg/L) Responses for Cu and Zn were then compared by analysing microwave spectral responses using a Lorentzian peak fitting function and investigating multi-peaks (peaks 0–6) and multi-peaks’ parameters (peak center, xc, FWHM, w, area, A, and height, H, of the peaks) for specific discrimination between these two similar toxic metals [74]. It is useful to compare additional parameters for determining the selectivity, as it was demonstrated by Harnsoongnoen et al. [57], to distinguish between sugars and salts. However, more work is required for reaching higher discrimination between similar contaminants.

**Figure 13:**
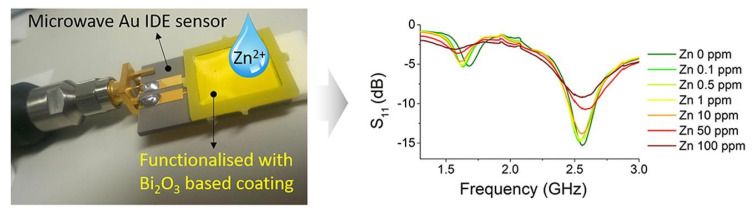
Microwave sensor functionalized with Bi_2_O_3_-based coating for the detection of Zn in water (**left**), and the dependence of the reflected signal (S_11_) response on Zn concentration (**right**). Reproduced with permission from [66] © Elsevier.

The microwave sensor functionalized with a 60-μm-thick β-Bi_2_O_3_-based film was developed specifically for the detection of Zn in water [66]. It showed an improved performance compared with the uncoated sensor and repeatedly detected the changes of Zn concentrations in water at 0–100 ppm levels with a linear response (Figure 13). Globally, Zn concentrations in mine water can be greater than 500 ppm, with typical concentrations ranging from 0.1 to 10 ppm. Thus, the proposed system can be adapted as a sensing platform for monitoring Zn in water in abandoned mining areas that would be able to detect unexpected events of pollution and to clarify metal dynamics. As recently reported by Vélez et al. [75], the f-EM sensor based on β-Bi_2_O_3_ is the one that exhibits the best performance (but a limited dynamic range) comparing glucose and NaCl, between others. Between the evaluated sensors, this sensor was the one that presented the highest sensitivity and the best resolution.

### 6.5. F-EM Sensors for On-Site and In Situ Applications

**Figure 14:**
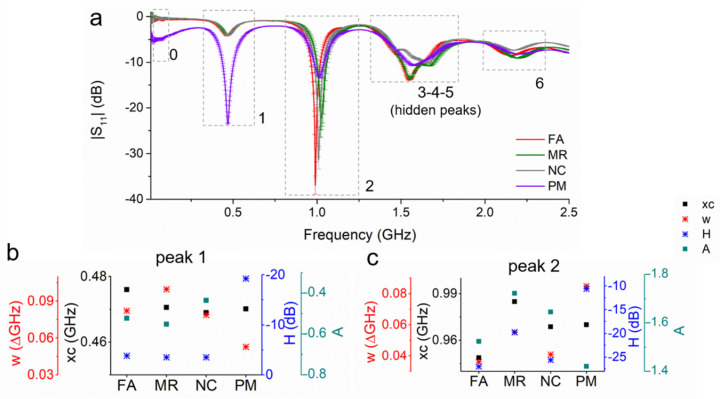
(**a**) Microwave spectral output for four polluted mining-impacted water samples collected in Wales (UK) analysed with an f-EM sensor based on L-CyCHBCZ coating and (**b**) peak parameters (w, xc, H and A) comparison for peaks 1 and (**c**) 2. Reproduced from [74], Creative Commons Attribution 4.0 International License.

The sensing system developed in [74] was tested on real water samples from two abandoned mining areas in Wales, UK, and evaluated the possibility of detecting both small and big differences between mining-impacted waters by comparing peak parameters in multiple peaks (Figure 14). Between the four samples, PM (a drainage adit from Parys Mountain mining district) was the most polluted (with 9.3 mg/L of Cu and 10.5 mg/L of Zn) compared to the other three samples (FA, MR and NC, from Wemyss mine) (with <0.001 mg/L of Cu and 2.9–9.3 mg/L of Zn). The sensor was able to distinguish between the two groups of samples in all the peaks, and at peaks 2–6 between the four samples, with both resonant frequency and amplitude shifts. Multiple peak characterization can give more information about the water composition. The sensor was able to perform a real-time identification of more- and less-polluted samples with high repeatability (with a coefficient of variation <0.05 dB), and evaluate changes in water composition. However, interferences were noticed, and more work is necessary for achieving higher selectivity.

Therefore, f-EM sensors can be considered a feasible option for real-time monitoring of water quality for a broad range of pollutants. Choosing the right coating or sensor functionalization allows for adapting the system to selected pollutants in a given scenario to ensure sensor response with high selectivity and sensitivity.

The sensing structure was adapted for directly probing the water, for then providing an *in situ* monitoring. Despite many recent technological advances and positive results obtained using this novel sensing system, significant work remains to be accomplished before a reliable smart sensor for water quality monitoring is achievable. Real mine water is more complex and characterized by high levels of dissolved metals and sulphate and, frequently, low pH. Though, there are obviously several challenges that must be overcome before this technology can ensure that measurements correctly identify distinct contaminants, and qualifying and quantifying the interferences caused by complex water matrices and similar pollutants.

## 7. New Trends in Water Quality Monitoring

The most interesting and recent efforts of online water quality monitoring rely on multisensor systems based on electrochemical methods along with advances in data processing and automatization of measurements.

A miniature device was reported in [76], where the authors combined a pH meter and a conductometer for evaluating drinking water quality. The system was tested over a period of 30 days in water streams of different speeds. It was shown that the device was working stably under these conditions. Though pH and conductivity are important water parameters, they are obviously not enough for comprehensive evaluation of water quality.

**Figure 15:**
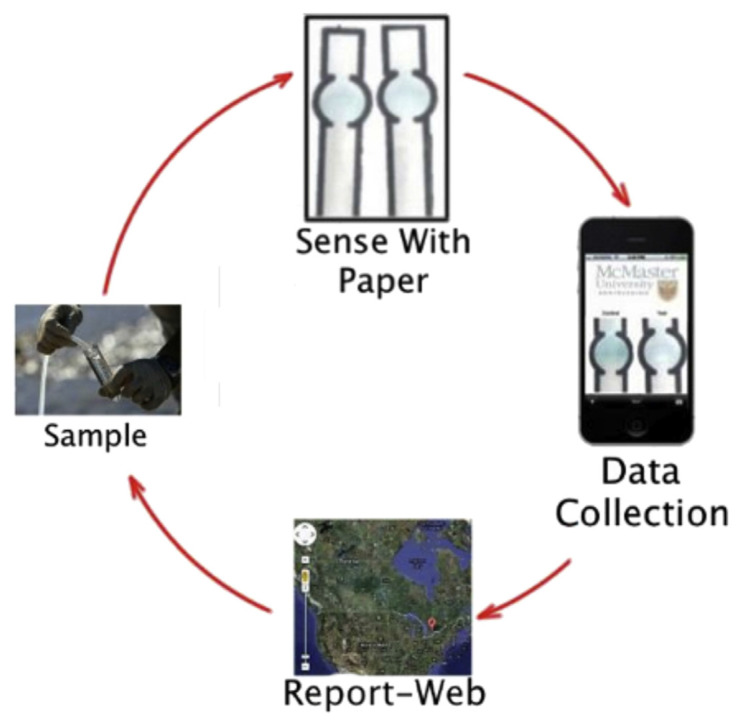
Concept of water quality evaluation using paper-based colorimetric sensors and a smartphone. Reprinted from Water Research, Vol. 70, Clemence Sicard, Chad Glen, Brandon Aubie, Dan Wallace, Sana Jahanshahi-Anbuhi, Kevin Pennings, Glen T. Daigger, Robert Pelton, John D. Brennan, Carlos D.M. Filipe, Tools for water quality monitoring and mapping using paper-based sensors and cell phones, Pages 360–369, Copyright (2015), with permission from Elsevier.

The demand for simplicity of the analysis is gaining momentum in the recent years. A combination of paper-based sensors and a smartphone application is described as an analytical instrument for water quality monitoring in [77]. The paper sensors generate colorimetric signals depending on the content of certain analytes and the cell phone captures such signals and compares them to that from a clean control sample. The smartphone would also transmit the results to the special site mapping the water quality. The schematic of this system is shown in Figure 15.

It was demonstrated that the system may quantify some organophosphorous pesticides like paraoxon and malathion in the natural water at the level 10^−8^–10^−6^ mol/L for both analytes. It is not clear however if such a device would perform correctly and precisely if multiple interfering species are present.

The successful application of a multisensor array for continuous online monitoring of processed water quality at an aeration plant was reported in [78]. The responses of 23 sensors of the array were continuously registered every seven seconds over a period of 26 days. The sensors were located in a special container with short direct connection to outlet water line of the aeration plant. The results of the multisensor were available immediately in a real-time mode. The use of topological data analysis (TDA) allowed for exploration of a very large dataset (295,828 measurements) accumulated through the whole period of continuous measurements. The achieved precision of analysis was suitable to monitor possible alarm events. No significant changes in water quality were observed over the experimental period. TDA helped visualizing some sharp changes related to sensor cleaning procedures and electrical power shortages.

**Figure 16:**
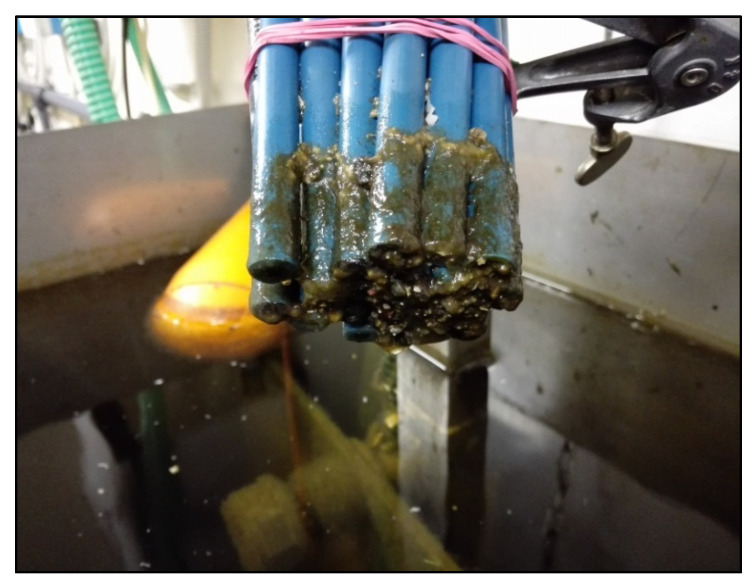
The sensor array after long-term online measurements at an aeration water plant. The sensors were cleaned by intensive washing. In spite of significant contamination, the sensors were stable for at least two months [79].

Figure 16 shows a multisensor system (MSS) applied to evaluate integral and discrete parameters of wastewater at two urban water treatment plants around St. Petersburg (Russia) [79]. A closely similar system was used for evaluation of the water quality from the Ganga river and city ponds in Kolkata (India). Good correlations (R^2^ = 0.85 for cross-validation) of MSS readings with COD values produced by laboratory chemical analysis were observed for all locations. This proves once again the applicability of MSS for real-time water quality analysis. A number of traditional water analytical parameters, such as ammonium and nitrate nitrogen and phosphorous were also determined with precision around 25% using the same MSS. All integral and discrete characteristics were calculated on the basis of the same set of measurements with MSS, without using any additional laboratory procedures, materials or qualified staff.

Along with traditional water quality analysis by analytical devices, a totally different approach has been developed. The global water toxicity (safety) can be also evaluated with the help of living creatures—from single-cell microorganisms to fishes, crustacea and mollusks. As a result, an ISO standard appeared in 1989, which suggested fresh water algae such as *Scenedesmus subspicatus* and *Selenastrum capricornutum* as a test. The decay of growth and reproduction of these bioassays is occurring in the contaminated water and can be used as its quality measure [80]. Another widespread method is based on the application of *Vibrio fischeri* bacteria exhibiting variations of luminescence dependent on water pollution [81]. Unfortunately, such methods have also been burdened by specific drawbacks such as the necessity to strictly maintain the livestock of biotests and realistic analysis times of at least 15–30 min (and up to few days) depending on bioassay applied. Therefore, these approaches can hardly be treated as online ones.

It must be noted that multisensor devices can also perform like biotests. The toxicity of polluted water samples was evaluated in [82] by a standard bioassay method and a potentiometric multisensor system comprising 23 cross-sensitive electrodes. Real wastewater samples from different regions in Catalonia (Spain) in addition to a set of model aqueous solutions of hazardous substances (54 samples in total) were used for the measurements. The obtained data set was treated by several regression algorithms; the results of the bioassay tests, expressed as EC50 (the concentration of sample causing a 50% luminescence reduction), were taken as Y-variable. The regression models were validated by full cross-validation and randomized test set selection. It was demonstrated that the proposed system was able to evaluate integral water toxicity with the errors of EC50 prediction from 20% to 25%. The suggested sensor array can be implemented in online mode, unlike bioassay techniques, which makes it a beneficial tool in industrial water quality monitoring.

## 8. Conclusions

The global need for developing novel platforms for real-time monitoring of various water pollutants is well-recognized. This paper provides a critical assessment of recent achievements in real-time water quality monitoring with chemical sensors in particular. The focus is given to those systems that were reportedly tested online, with real water samples and their feasibility for long-term use is considered. The review shows that there are still many obstacles for having one sensing approach that would satisfy different situations. The most successful systems based on chemical sensing or its combination with other methods rely on specificity of a coating material that is capable of accurate detection of certain water pollutants, with molecularly imprinted polymers providing an increased flexibility for the designing of those systems.

## Figures and Tables

**Table 1:** Comparison of statistical features between uncoated and coated sensors for a water sample collected in a mining area spiked with Cu using the standard addition method. Reproduced from [74], Creative Commons Attribution 4.0 International License. LoD: limit of detection.

	*R^2^*		*CV* *(*dB*)*		*Sensitivity* *(*ΔdB/mg/L*)*		*LoD* *(*mg/L*)*		*Q-Factor*	
	UNC	f-EM	UNC	f-EM	UNC	f-EM	UNC	f-EM	UNC	f-EM
**Peak 0**	0.970	0.928	0.20	0.25	0.362	0.222	0.194	0.379	/	/
**Peak 1**	0.963	0.981	0.02	0.03	0.354	0.260	0.146	0.409	2.60	6.57
**Peak 2**	0.888	**0.983**	0.01	0.02	0.824	**1.651**	0.083	**0.036**	30.71	**135.48**
